# Maternal Impressions

**Published:** 1881-11

**Authors:** J. Davis


### MATERNAL IMPRESSIONS.

*Ed. Med. and Sur. Rep*:—

Recently there has been a good deal said through the columns of the Reporter, about maternal impressions, and with your permission I will refer to a few cases:—

The first case, a Mrs. R.: during her pregnancy one of her sons got into a fight and received a knife wound across his wrist, severing the softer parts, to the bone; in this condition the boy was received and wound dressed by his mother; and when the child was born it had a like gash across its forearm, which resisted all efforts at healing until the hand and arm above the wound were taken off; the stump then readily healed.

The second case was a Mrs. T., of Dewiess County, who, while pregnant, attended a young lamb that had its navel torn by some pigs; she tried to cure the torn cord, and the child which was afterwards born to her had a bleeding umbilicus which refused to heal, and at ten years old the otherwise stout boy had a lacerated and running navel. J. DAVIS, M. D.

*Nevada, Mo.*

